# Dentin bonding agent with improved bond strength to dentin through incorporation of sepiolite nanoparticles

**DOI:** 10.4317/jced.53722

**Published:** 2017-06-01

**Authors:** Farnoosh Fallahzadeh, Shadab Safarzadeh-Khosroshahi, Mohammad Atai

**Affiliations:** 1 Assistant Professor of Operative Dentistry, Dental Caries Prevention Research Center, Qazvin University of Medical Sciences, Qazvin, Iran; 2 Professor, Department of Polymer Science, Iran Polymer and Petrochemical Institute (IPPI), Tehran, Iran

## Abstract

**Background:**

The study aims to investigate the effect of incorporation of sepiolite nanoparticles on the microtensile bond strength of an experimental dentin bonding to the human dentin.

**Material and Methods:**

The sepiolite nanoparticles were incorporated into an experimental methacrylate-based dentin bonding system in concentrations of 0.0, 0.2, 1.0, 2.0, and 5.0 weight percents. The specimens were then ultrasonicated to finely disperse the nanoparticles in the adhesive matrix. The coronal enamel of 30 intact human premolars was cut to expose dentin. Having etched, rinsed, and blot dried the experimental bonding agents were applied to dentin surface. Adper Single Bond was used as control group according to the manufactor’s instruction. Then all the teeth were built up by composite and sectioned in stick form for microtensile test. The fracture surface was observed using SEM. The data were analyzed by ANOVA and Tukey’s post-Hoc test.

**Results:**

The results indicated that the incorporation of the nanofiller, improved the bond strength to dentin with the highest values obtained at 1 w% sepiolite nanoparticle content.

**Conclusions:**

Sepiolite nanoparticles can be considered as novel fillers to improve the mechanical properties of dentin bonding agents.

** Key words:**Dentin bonding agent, nanoparticles, bond strength, sepiolite, microtensile test.

## Introduction

The most important aim of using dental adhesives is to bond dental restorative materials to tooth structure (1). Adhesion to tooth structure prevents postoperative sensitivity, secondary caries, discoloration and microleakage (2). Because of dynamic and hydrated nature of dentin, bonding to enamel is more durable than dentin (3,4). Hybrid layer which forms by polymerized bonding monomers penetrated into dentin structures, is the basis of dentin adhesion (5,6). Among resin-dentin components, adhesive layer has the lowest elastic modulus. During stress application on resin-dentin complex, adhesive layer shows the greatest level of strain (7). Stress concentration on this weakest layer during occlusal loading or composite polymerization, may cause defects, cracks or resin-dentin bond failure (8,9).

It has been suggested that incorporation of fillers into dental adhesives would enhance the mechanical properties of the adhesive layer (4,10-12). Filled adhesives improve mechanical strength by shock-absorbing effect (7,13).

Sepiolite, a nanoclay with the formula of Mg8Si12O30(OH)4(H2O)4•8H2O is a needle-like structure which is based on the units of phyllosilicates: silica tetrahedral and Mg2+ or Al3+ octahedral (14,15). Its properties provide solutions for applications ranging from carrier for chemicals, as a rheological additive for industrial paints, as processing aids, and binding additives. But a very new application is the use as nanofillers in polymer systems (14). Because of the great number of active centers on its surface (silanol groups and Mg2+-coordinated water), sepiolite has a high potential interaction level between both nanofillers-nanofillers and nanofillers-matrix components. This excellent adhesion/compatibility with polymeric matrices and the strong anisotropy of this mineral material provides the excellent reinforcing effect on polymers, increasing the mechanical properties of the final compounds (15).

In this study the sepiolite nanoparticles were incorporated into an experimental dentin bonding as reinforcing filler. The bond strength of the bonding agents to the human dentin was then evaluated by microtensile test.

## Material and Methods

This study has been reviewed by the Medical Research Ethical Committee of the Qazvin University of Medical Sciences No: IR.QUMS.REC.1394.370 and there is no conflict with ethical considerations. 2-Hydroxyethyl methacrylate (HEMA), camphor-quinone (CQ), 2-ethyl-2-hydroxymethyl-1,3-propandiol trimethacrylate (TMPTMA), and ethanol were purchased from Merck (Germany). N,N-Dimethylaminoethyl methacrylate (DMAEMA) were obtained from Fluka (Germany). 2,2-Bis[4-(2-hydroxy-methacryloxypropoxy)phenyl] propane(Bis-GMA) and Methacryloxypropyl trimethoxysilane (γ–MPS) were kindly provided by Evonik (Germany). AdperTM Single Bond2, a commercially available nanoparticle containing dentin bonding, was obtained from 3M ESPE (USA). The 37% phosphoric acid gel (Gel Etchant) was obtained from3M ESPE (USA). Pristine sepiolite (Pangel S9) was kindly provided by Tolsa SA (Spain). The specific surface area of the sepiolite is 320 m2g-1 (BET method, N2 adsorption) (15).

-Preparation of sepiolite nanoparticles

A solution consist of ethanol (70 w%) and distilled water (30 w%) was prepared. The pH of the solution was adjusted at 5 by adding a few droplets of acetic acid. γ–MPS was added to the solution (20 wt% based on the filler) and left for 1 hour to be pre-hydrolized. The sepiolite was added to the solution and sonicated for 2 minutes using a probe sonicator (Bandelin, Germany). The suspension was left for 1 day for completion of the condensation reaction of silane coupling with the silanol groups on the sepiolite surface. The silanized filler was then centrifuged and separated. Having washed several times with ethanol/water (70/30 wt%/wt%), the nanoparticles were dried and ball-milled.

-Preparation of adhesive

The adhesive was prepared according to the formulation shown in table 1. Then, the silanized sepiolite were added to the adhesive in 0, 0.2, 1, 2 and 5 wt.%. The fillers were well dispersed in the adhesive solution by ultra-sonication using the probe sonication apparatus for 2 min in an ice bath. 0.5 wt.% CQ and 1 wt.% DMAEMA as photoinitiator system were then added into the adhesive.

-Teeth preparation

Thirty human premolar intact teeth that were extracted for orthodontic treatments were used in this study within 6 months of extraction. After removing soft tissue debris, they were immersed in a 0.5 w% chloramine-T solution for 1 week and then stored in distilled water in a refrigerator at 4°C. The coronal enamel was removed with a low speed diamond saw (ref.070 D&Z ,Germany) to form a flat dentin surface. The dentin surface was then polished with a 600-grit silicon carbide abrasive (paper Soft flex 991 A, Germany) under running water to create a uniform smear layer. The teeth were randomly assigned to six groups, six teeth in each group. For groups one to five, the flat occlusal dentin surface was etched with a 37% phosphoric acid etching gel (3M ESPE,USA) for 15 s-and rinsed with distilled water for 15s. To achieve wet bonding technique, excess water was blot dried with tissue paper. The experimental one-bottle adhesive was applied in two layers with a microbrush using agitating motions. After 15 s application, the surface was gently dried with oil and dust-free air for 5 s. After confirming the glossy appearance of the entire dentin surface, the adhesive was cured for 20s. In the sixth group, after applying phosphoric acid, AdperTM Single Bond Plus 2 (3M, ESPE,USA), was applied on dentin according to the manufacturer’s instructions. A restorative composite (Z 250, shade A2; 3M ESPE, USA) was built-up on the adhesive-treated surfaces up to 5 mm thick by incremental technique. Each increment was 1mm and cured for 40s (OPTILUX 501, Kerr, USA) with the intensity of 600 mW/cm2. The bonded specimens that mounted in an acrylic mold, were sectioned into bar shaped specimens with 1mm×1 mm cross-section areas at bonding-dentin interface using a low-speed diamond saw (Isomet, Buehler Ltd., Lake Bluff, IL 60044,USA).

-Microtensile bond strength test

After 24 h storage in distilled water at 37°C, the specimens were attached to the fixture of a universal testing machine (STM 20, Santam, Iran) with a cyanoacrylate glue and subjected to microtensile bond strength (µTBS) testing at a crosshead speed of 0.5 mmmin-1 until they fractured. Data were collected and the microtensile bond strength was then calculated by dividing the force at break by the composite-dentin interface area.

-Scanning electron microscopy

The fractured composite-dentin interfaces, after gold sputter coating, were observed with a scanning electron microscopy (EMI-TECH, SC760, UK).

-Statistical analysis

The results were analyzed and compared using one-way ANOVA and Tukey’s post-Hoc test at the significance level of 0.05. Analysis was performed by SPSS statistical software (SPSS 15.0 for Windows).

## Results

**Figure 1:**
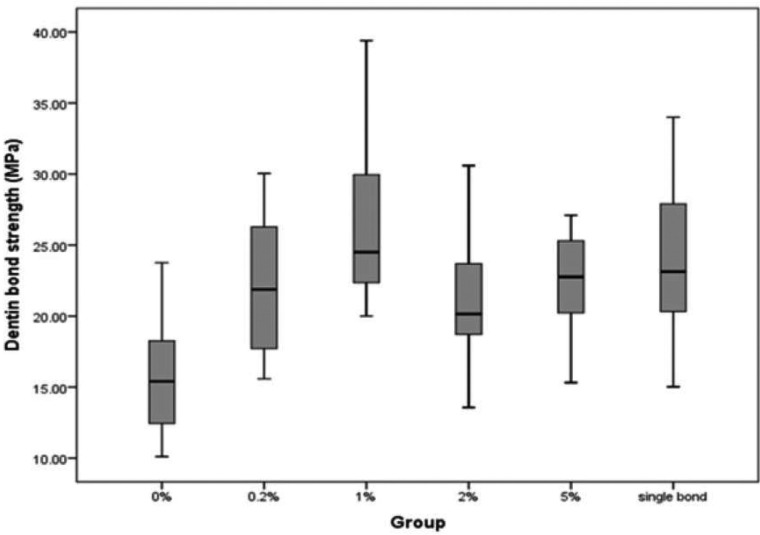
Microtensile bond strength to dentin of the experimental dentin bondings and the control group.

One-way ANOVA showed a significant difference between the µTBS of six groups (*P*<0.05) (Fig. 1).

Multiple comparisons (post hoc Tukey’s test) revealed that µTBS in first group (0 wt%) was the lowest and in the third group (1% wt) was higher than second (0.2 wt%) and fourth group (2 wt% ) significantly (*P*<0.05).

Other groups were not significantly different (*P*>0.05). The third group (1wt%) had the highest µTBS. Although the µTBS of the control group (Adper Single Bond Plus 2) was lower than third group, the difference was not significant (*P*>0.05).

**Figure 2:**
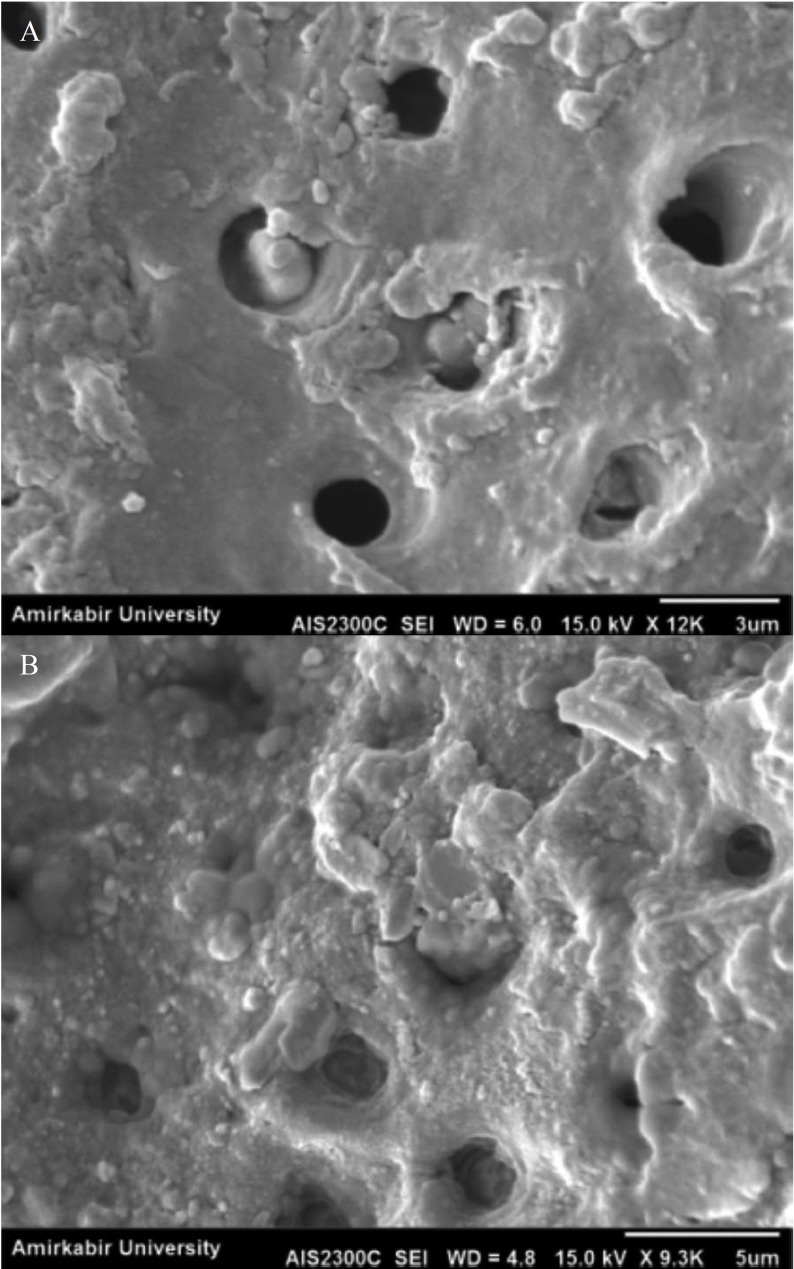
SEM micrographs of the adhesive containing: (A) 0 wt % and (B) 1 wt% sepiolite nanoparticles.

SEM observations of the resin-dentin interfaces revealed that most of the tubules were filled by adhesive in third group (1 w%t) whereas in first group (0 wt%) most of them were empty (Fig. 2).

## Discussion

Complexity of oral environment causes bonded interface degradation over the time (16). Bonding to dentin is less durable than enamel (17). Results of this study revealed that the incorporation of the sepiolite nanoparticles increases microtensile dentin bond strength. The mean bond strength of group A (experimental adhesive containing no nanoparticles) was significantly lower than the other groups. It has been shown that the incorporation of nanoparticles into the dentin bonding agents resulted in improved mechanical properties and improved bond strength to dentin (4,10-12). Filled adhesives act as an intermediate shock-absorbing elastic layer between composite and dentin bonding, so increases the bond strength to dentin (18). The intermediate layer and the resin-impregnated dentin, form an elastic buffer which offers the resin–dentin interface a sufficient strain capacity to accommodate both the composite and dentin (17).

Amoung the groups,the adhesive containing 1 wt% sepiolite nanofiller showed the highest microtensile bond strength (26.4 MPa). Kasraei *et al.* (19). reported that nanosilica filled adhesive containing 1wt% showed the highest bond strength. Kim *et al.* (7) also reported that silica nanofiller containing adhesive, exhibited the highest microtensile bond strength at 1wt%. They stated that incorporation of nanofillers greater than interfibrillar space (20 nm) not only increases the viscosity, but also causes the agglomeration of filler contents on dentin surface.

Further increase in filler content, resulted in a decrease in bond strength. Miccrotensile bond strength decrease was attributed to the agglomeration of the nano-particles at higher filler contents. Very small nanofillers easily aggregate because of their high surface energy (7). The accumulation of these filler agglomerates on the top of the etched dentin substrate, prevent the penetration of adhesive monomers into the dentinal tubules and hybrid layer. Another factor for decreasing the penetration of resin adhesive is the higher viscosity of adhesive at higher filler content. These result in decreasing the micromechanical retention which is the most important factor in dentin bond strength.

Other studies showed higher strength at lower filler contents. Solhi *et al.* (10) found the highest microshear bond strength of ad-hesive containing PAA-g-nanoclay at 0.2wt%. They suggested that the adhesive containing 0.2wt.% penetrated into tubules providing micromechanical interlocking by the resin tag formation, whereas in adhesive containing higher filler content (5 wt.%) the penetration is not complete and leaves most of the tubules empty. The lack of resin tags results in a poor retention and a gradually drop in microshear bond strength.

Lohbauer *et al.* (20) reported the highest bond strength in 20wt% filler added to primer of a three step bonding agent. He suggested that the spherical shape of the nanoparticles by using laser vaporization of the raw zirconia powder provides particles with only one point of contact, so decreasing the tendency of agglomeration, cause a small surface area available for particle–particle attraction and less energy is needed to break these interactions. Also this higher content was added to primer (not adhesive) which maybe because of lower viscosity of primer, addition of higher filler is more possible.

Decreasing bond strength at higher filler content was also reported in the other studies (4,7,10-12). This decrease is attributed the agglomeration of very small nanoparticles, above the etched dentin surface. In this condition, demineralizad dentin acts as a cribriform and prevents resin infilteration into dentin (10,13,21). Agglomerated fillers form clusters and these agglomerated clusters make flaws which concentrate the applied stress leading to decrease in the bond strength (4,7,13).

In this study the group with 1wt% filler showed non-significant higher bond strength than control group (Adper Single Bond Plus 2,3M ESPE). Adper Single Bond Plus 2 is a single bottle fifth generation consists of silica nanofillers. In single bottle systems silica fillers are also used as a viscosity and thickness enhancement agent to prevent overthining and lacking of polymerization due to oxygen-inhibition (22-25).

## Conclusions

The results of this study showed that incorporation of sepiolite nanoparticles into an experimental dentin bonding system enhanced the microtensile bond strength to human dentin. The highest increased was found at 1 wt%. The results indicate that the sepiolite nanoparticles are promising reinforcing fillers to improve the bonding strength of dentin bonding agents.
